# Observing Live Fish Improves Perceptions of Mood, Relaxation and Anxiety, But Does Not Consistently Alter Heart Rate or Heart Rate Variability

**DOI:** 10.3390/ijerph16173113

**Published:** 2019-08-27

**Authors:** Nancy R. Gee, Taylor Reed, April Whiting, Erika Friedmann, Donna Snellgrove, Katherine A. Sloman

**Affiliations:** 1 Department of Psychology, State University of New York, Fredonia, NY 14063, USA; 2 WALTHAM Centre for Pet Nutrition, Leicestershire LE14 4RT, UK; 3 Department of Psychiatry, School of Medicine, Virginia Commonwealth University, Richmond, VA 23298-0710, USA; 4 School of Nursing, University of Maryland, Baltimore, MD 21201, USA; 5 Institute of Biomedical and Environmental Health Research, School of Health and Life Sciences, University of the West of Scotland, Paisley PA1 2BE, UK

**Keywords:** human-animal interactions, health benefits of companion animals, psychological benefits of aquarium fish

## Abstract

Although fish and other aquatic species are popular privately-kept pets, little is known about the effects of watching live fish on the perceptions of arousal and the link between those perceptions and physiological measures of arousal. In two separate experiments, participants were asked to watch identically-equipped fish tanks for five minutes in each of three conditions: (1) Live fish, (2) plants and water, and (3) empty tank. Linear mixed models used across both experiments revealed similar results: Greater perceptions of relaxation and mood, and less anxiety during or after viewing the live fish condition, compared with the other conditions. Heart rate and heart rate variability responded to the arousal associated with a math task, but did not differ consistently across viewing conditions. These results suggest that the link between perceptions of arousal, and the physiological measures associated with arousal, may not be strong or immediate, or that heart rate and heart rate variability may not be appropriate measures for the test population. Implications of these results for the biophilia hypothesis and the biopsychosocial model are discussed.

## 1. Introduction

Aquaria containing fish and other aquatic animals are commonly found in homes, offices, assisted living facilities and classrooms. In the US, approximately 12.5 million households include freshwater fish aquaria, and an additional 2.5 million include saltwater aquaria [1], making fish the third most popular pet behind dogs (in 60.2 million households) and cats (in 47.1 million households). As an aquarium will generally contain more than one individual, the total number of fishes owned is estimated to be as high as 139.3 million freshwater, and 18.8 million saltwater, fish, eclipsing cats (94.2 million) and dogs (89.7 million). However, most research examining human and companion animal interactions focuses primarily upon dogs [2], followed by horses, cats and other mammals (e.g., guinea pigs). 

Mounting evidence shows health benefits to humans related to companion animal ownership. For example, a study comparing new pet owners to non-owners found that those who acquired a pet experienced a number of reductions in minor health problems in the first few months of ownership, and new dog owners increased their recreational walking for an extended period of time [3].

Generally, dog owners are more physically active than non-owners, and are more likely to achieve recommended levels of physical activity [4]. Dog ownership was causally linked to a reduced risk of cardiovascular disease in their owners by the American Heart Association [5]. Interacting with a companion animal has also been linked to a number of other positive health benefits for humans, such as lower mortality [6], reduced loneliness and decreased social isolation [7], lessened Post Traumatic Stress Disorder (PTSD) symptoms [8], and diminished anxiety [9], and to increased social interactions [10], language output and verbal fluency [11].

It is important to note that while these and other findings indicate positive effects of companion animal ownership or interaction, there are also a number of studies reporting null results or negative findings. More research is needed to clarify what is effective, when it works, and how it works. Additionally, the study of Human Animal Interactions (“HAI“) could benefit from the development and use of a unifying theory to explain results and guide future hypothesis development and testing.

The concept of biophilia [12] is frequently called upon to provide a partial explanation of the beneficial effects associated with HAI. Wilson (1984) described biophilia as humans’ “innate tendency to focus on life and lifelike processes”. The evidence of human interest in and interaction with nature appears in the form of national parks, zoos and aquaria, public and private botanical gardens, and the practice of keeping indoor plants and pets at home [13] People also travel long distances to be in, and feel closer to, nature, and there is some evidence to indicate that being in the presence of nature promotes physical and psychological well-being, while decreasing everyday stress [13]. Historically, humans have been drawn to bodies of water, forests and other landscapes that provide sustenance, shelter and protection [13]. Our (theoretical) evolutionary background as hunter-gatherers may also explain the human tendency to be drawn to other animals and to identify them as animals that are useful, safe or potentially threatening [14].

The positive effect of companion animals also may derive from distraction, as attention to an animal draws attention away from perceived stressors [14]. In previous research children preferentially attended to [15], chose to interact with [16] and better categorized [17], living things compared to non-living things. These results suggest that animals are effective at capturing our attention, but a number of other unique experiences (such as viewing an impressive scenic vista such as Niagara Falls) also tend to capture our attention. This leaves us to wonder what, if anything, is special about living animals that may confer a variety of health benefits to humans?

The biopsychosocial model provides a framework for understanding the impact of HAI on human health [18]. In this model health is determined by contributions from biological, psychological and social factors, all of which interact with one another. A disruption or enhancement in one of those factors can affect the other two, and impact an individual’s entire state of health. Companion animals may contribute to human health by enhancing these three factors in multiple ways. They are thought to provide social support, via companionship and an opportunity to nurture, which in turn reduces loneliness and depression. They provide opportunities for exercise via dog walking and general pet care. Some animals, particularly dogs, increase feelings of safety, reducing anxiety and stress. Companion animals may also help to buffer the impact of disruptions to any of the three factors [5]. For example, suffering the loss of a loved one has been associated with shorter periods of bereavement in older adults who own a pet, compared to those who do not [19].

While the vast majority of HAI research has focused on mammals, there has been little research into the effects of owning or viewing aquatic animals [20]. Although limited, the available research does show beneficial effects of viewing fish in aquaria. For example, a study of older adults watching either live fish in an aquarium or a similar videotape found a decrease in the physiological markers of stress [21] when compared with those watching a placebo videotape, while in another study of older adults with dementia, eating in the presence of an aquarium increased food intake [22]. Anxiety, as measured by the State Trait Anxiety Inventory—Short form (STAI-S), was significantly reduced when university students who were about to give a speech were given the opportunity to watch a fish tank for 5 min compared to the control condition [23]. 

In an examination of blood pressure (BP), university students watched videos of fish in an aquarium, birds in an aviary, primates in a zoo or control videos of a popular soap opera or a blank screen, and then completed a reading aloud task [24]. All animal videos produced significantly lower heart rate (HR), and systolic and diastolic BP, compared to the control videos. Another study examined psychological and physiological responses when viewing un-stocked, partially-stocked and fully-stocked aquarium exhibits [25]. Participants became more relaxed when viewing the aquaria, and wanted to view the partially- and fully-stocked aquaria for longer amounts of time. In one study involving both individuals with BP at the low end of normal and others diagnosed with hypertension, BP fell while viewing a stocked fish tank after having been artificially raised by a stress-inducing activity. The level of reduction in both systolic and diastolic BP was most pronounced in the hypertensives, to the point of clinical significance [26].

The literature is not wholly conclusive with regards to the impact of viewing fish in aquaria. For example, Barker and colleagues [27] randomly assigned patients awaiting electroconvulsive therapy treatment to wait either in a room that had an aquarium, or a similar room that did not. The only finding that approached significance was a trend towards a reduction in anxiety (*p* = 0.08) for the aquarium group compared to the no-aquarium group; no results were found in measures of depression, fear and frustration. In another study conducted in a hospital setting, aquaria were installed in the rooms of patients awaiting heart transplants [28]. Anxiety, depression, hostility, dysphoria and sensation seeking and positive affects were assessed at baseline, after three days, and after 11 days. No significant differences were reported at any of the assessment periods.

Considering the results presented above, it may be the case that viewing fish can moderate the impact of stress when the stress level is not high, or the participants are not already health-compromised in some way, as is the case for patients awaiting ECT or a heart transplant. It also may be that prolonged sessions of viewing an aquarium with fish in it leads to longer attention on it, and may lead to different responses than viewing it for only a short time. In order to detect any stress-reducing or moderating impact of viewing a fish tank, it may be important to place the participants in a low or moderate level of stress, rather than a high state of stress. While university students are under stress, especially related to transitioning from high school to college [29], they are generally considered to be a healthy population. Thus, they may represent an ideal population in which to examine the stress, or anxiety, reducing the impact of viewing live fish.

Observing fish is a very different style of human-animal interaction as compared to interacting with other mammals. There is no way to safely hold or pet a fish; rather, one must observe the fish in an aquarium or another body of water. This does not mean, however, that humans do not form connections to these fish. Indeed, a large number of individuals who own fish as pets become very attached to them, and along with the worldwide popularity of public aquaria, this indicates that fish fall well within the human attraction to nature as posited by the biophilia thesis. Pet attachment, typically examined with mammals, has been suggested to have numerous health benefits which include child and adolescent development, increased caring and compassion, and even improved immune system function [30], making it important to examine whether attachment to fish plays a role in any health-related benefits of viewing fish in aquaria. The current study involves university students viewing three different aquarium conditions: An empty tank, a tank with water and plants, and a tank with water, plants and live fish.

Because we recognized in advance that a study involving self-report measures is subject to demand characteristics, we chose to include physiological measures in the form of Heart Rate Variability [HRV] and Heart Rate [HR] as well. HR and HRV both have been shown to be effective indicators of stress and anxiety [31,32].

According to the concept of biophilia, humans have an innate preference for life and nature. For this reason we predict that the tank containing all three elements of fish, plants and water should produce the most pronounced results, both self-reported and physiologically tested. 

We predict the highest levels of relaxation, mood and Heart Rate Variability (HRV—indicative of physiological relaxation) and the lowest levels of anxiety and Heart Rate (HR—indicative of physiological arousal) in the live fish condition, followed by the aquarium with plants and water, which should be followed by the empty aquarium.

The current study also includes a stressor in the form of a math task to be completed at the end of each aquarium viewing condition. Based on previous research, we predicted that the math task would increase arousal as demonstrated by a higher HR and lower HRV, as compared to the measures taken during the preceding aquarium viewing conditions. We also predicted that this increased arousal would be moderated or buffered in the live fish condition relative to the empty tank condition, and that the condition which includes water and plants but no fish will be somewhere between. A series of studies demonstrated that the presence of a companion animal can buffer a stress response (for a summary of this work see Friedmann & Gee, 2017) [33], and based on this research we predict that viewing live fish will have similar effect.

## 2. Experiment 1

### 2.1. Method

#### 2.1.1. Participants

A priori sample size calculations indicated a sample size of 35 would provide a power of 0.80 with alpha 0.05, correlations among repeated measures of 0.30, and a medium effect size. Therefore, data were collected and used from 35 undergraduate students currently enrolled at the State University of New York at Fredonia. Three participants were male, 31 participants were female, and one participant identified as neither male nor female. We gathered a variety of demographic characteristics from our participants to better describe our sample, but did not use this information in further statistical analyses. Three participants recorded having an allergy to dogs, while nine participants recorded having an allergy to cats. Thirty participants self-identified as Caucasian, four identified as African American, three identified as Latino, and one participant identified as an American Indian. Ages ranged from 18 to 24 years old (*M* = 19.38, *SD* = 1.23). Participants included six freshman, 23 sophomores, five juniors and one senior. Other characteristics of our sample included average GPA on a 0-to-4 scale GPA (*M* = 3.06, *SD* = 1.37), older siblings (*M* = 1.66, *SD* = 2.34), younger siblings (*M* = 1.00, *SD* = 0.97), and even the number of close friends (*M* = 2.71, *SD* = 1.02) was reported. Three participants currently resided with a pet at the time of the study; two were the owners of the given pet, one was not. Twenty-two participants recorded that they were the owner of a pet that does not currently reside with them. Of those, 13 participants indicated that their pets did not currently live with them because their landlord or housing policies did not allow pets, two did not have time to care for their pets, three knew their pets would not benefit from living with them in their current living situation, one said that they did not have the proper transportation to bring that pet to their current residence, and three listed reasons other than the provided options. The pet owners in this sample reported moderately high pet attachment as measured by the Lexington Attachment to Pets Scale (“LAPS”) [34] (*M* = 48.60, *SD* = 7.66). This scale has 23 items, with high scores indicating greater attachment (highest possible score = 69, some items were reverse scored). Although the number of participants in both Experiment 1 and Experiment 2 who reported owning pets is somewhat above the 67% of households cited by the American Pet Products Association [35], the participant percentage is somewhat misleading, as some of the demographic questions in these studies were not mutually exclusive. For instance, an individual who reported that they own pets who currently live with them, may also have indicated that they own pets who do not live with them. Thus, it is unlikely that the experiments reported here were unduly biased in favor of pet owners.

#### 2.1.2. Materials

A demographic questionnaire requested the information reported above. Self-identified (current or previous) pet owners also completed the LAPS. Participants were also given pet experience and pet history questionnaires that included questions about the information reported above. Participants who did not own a pet were also asked questions about their reasons for not owning a pet, if they have ever owned a pet, and what type, the length since their last pet ownership, and their attachment toward their last pet(s). Additionally, non-pet owners were asked questions about any regular contact they have with other pets, what type of pet(s) they have regular contact with, if they helped to care for any pets, and if so, how frequently they cared for someone else’s pet. Also used in the study were mood and relaxation scales, both of which were scored on a 7-point Likert scale. The mood scale ranged from Extremely Unhappy (1), to Neutral (4), to Extremely Happy (7). Positioned above the Likert scale were emoticons ranging from frown to neutral to smile. The relaxation scale ranged from Not at All Relaxed (1), to Moderately Relaxed (4), to Completely Relaxed (7). The math task included simple math problems in addition, subtraction, multiplication and division. All math items were presented to the participants at one time on multiple sheets of paper, with each page containing 36 problems. Participants were free to answer as many problems as they could, in any order, during the time allotted. None of the participants were able to complete all of the problems.

#### 2.1.3. Aquaria and Apparatus

Three 20-gallon Aqueon fish aquaria were used in the study, and all were set up with identical décor, equipment and black backgrounds. All three aquaria were filtered by AquaClear 30 filters, contained individual heaters, sponge filters and thermometers, and were decorated with a tan decorative arch, a small ceramic cave, and 20 pounds of neutral-colored substrate covering the bottom. Other than the equipment and décor described above, one aquarium, the empty tank condition, was not otherwise altered. The other two aquaria were filled with dechlorinated water, and 15 small Amazon Sword (*Echinodorus bleheri*) plants (approximately three to eight inches tall) were planted in similar locations in each tank. The tank with only these enhancements is referred to as the plants and water tank. Fish were added to the last tank, which is referred to as the live fish tank. This tank included one albino bristle nose Plecostomus (*Ancistrus temminckii*), twenty male fancy guppies (*Poecilia reticulata*), and one blue veil tail betta (*Betta splendens*).

Also used in the study was the Polar Pro Trainer RS800CX heart monitor, which measured both Heart Rate Variability (HRV) and Heart Rate (HR). The heart rate monitor utilized a chest strap worn by participants that transmitted data via blue tooth to a receiver for capture and later analysis. Polar wireless heart rate monitors are reliable during physical activity in human participants [36].

### 2.2. Procedure

The study was conducted in accordance with the Declaration of Helsinki, its protocol approved by the State University of New York Fredonia Institutional Review Board (IRB) on 4 October 2018 (approval period extended on 7 March 2019) and identified as: “Investigation of the Impact of the Presence of Live Fish on Human Psychological and Physiological Responses”. The project was declared exempt by the State University of New York Fredonia Animal Care and Use Committee (ACCU) on 2 October 2018. Data collection did not commence until after approvals were received.

All subjects gave their informed consent for inclusion before they participated in the study. Upon arrival at the laboratory, the participants were asked to read and sign a consent form, and they were verbally asked about their current pet ownership status so they could complete the appropriate pet ownership questionnaire (e.g., pet owner or non-owner). Following the pet ownership questionnaire, self-identified pet owners also completed a pet attachment questionnaire. All participants completed the pet ownership history and experience questionnaire, the demographic questionnaire, and the mood and relaxation scales. After the participants completed the paperwork, they were asked to put on the HRV monitor strap. 

The experimenter checked that the monitor was transmitting consistently, and the participant was assigned in randomized block fashion to complete each of the three tank conditions: Live fish, plants and water, and empty tank, according to the order assigned to them prior to their arrival at the laboratory.

Based upon the random assignment procedure, the participants were taken into the first tank viewing area, located behind a room divider, such that they could not view the other conditions. They were asked to sit quietly and watch the aquarium for five minutes. Upon conclusion, they were asked to complete the mood and relaxation scale, and then they were asked to complete the timed math task described above within two minutes, completing the problems in any order. A researcher timed their progress and asked them to stop at the end of two minutes. They were then asked to complete the mood and relaxation scales again. This process was repeated for the other two remaining tank viewing conditions.

### 2.3. Results

#### 2.3.1. Statistical Methods for both Experiments

Random intercept Linear Mixed Models (LMM) were used with all measurements nested within participants. Inter Class Correlations (ICC) were calculated and revealed interdependence among measurements greater than 30% for each outcome. After the unconditional mean models, each set of models began with a factorial model including condition (live fish, plants and water, or empty tank), activity (viewing tank or performing math task), and running order, and included all two-way interactions and the three-way interaction. Best models were identified via examining Akaike information criterion (AIC) and Bayesian information criterion (BIC) in an iterative process of removing variables from the original models. A separate series of models were run for each outcome (HR—Heart Rate, HRV—Heart Rate Variability as measured by the root mean square of successive differences (RMSSD), relaxation score, mood score (Experiment 1 only), and State Trait Anxiety Inventory (STAI—Experiment 2 only)). Significance of differences according to categorical variables were investigated using pairwise comparisons with corrections (Sidak) for multiple comparisons.

Prior to multivariable analyses, descriptive statistics were calculated, data were cleaned (this process removes erroneous readings related to participant movements, such as adjusting their body position or sneezing, recorded by the HRV monitor), and assumptions tested. Normality was examined for each outcome, and transformations required for HRV and HR. The best transformation was identified based on skew and QQ plots. All analyses were run with SPSS 25. Statistical significance was set at *p* < 0.05, and comparisons were two-tailed.

#### 2.3.2. Experiment 1 Results

The best models for all outcomes included the main effects of activity, condition and running order. No two- or three-way interactions remained in the models.

##### HRV

The HRV data were transformed using a square root transformation. The only significant independent effect was for activity [*F* (1, 169.053) = 5.975, *p* = 0.016], showing that the HRV was lower while participants were doing the math task (*M* = 5.200, *SE* = 0.313) than while they viewed the aquaria (*M* = 5.592, *SE* = 0.314; *p* = 0.016), controlling for condition and order.

##### HR

The HR data were winsorized at 100. Activity significantly independently predicted HR [*F* (1, 169.717) = 4.229, *p* = 0.041]. HR was higher while participants were doing the math task (*M* = 84.256, *SE* = 1.631) than while they viewed the aquaria (*M* = 82.458, *SE* = 1.632; *p* = 0.041). Condition also significantly independently predicted HR [*F* (2, 161.910) = 5.153, *p* = 0.007]. 

After controlling for (including in the model) running order and activity, HR was lower while participants viewed the tank containing live fish (*M* = 81.849, *SE* = 1.687) than when they viewed the tank containing plants and water (*M* = 85.242, *SE* = 1.687; *p* = 0.006]. No other differences were statistically significant.

##### Relaxation Score

Activity significantly independently predicted the relaxation score [*F* (1, 169.194) = 41.992, *p* < 0.001]. Relaxation was rated higher after participants viewed the aquaria (*M* = 5.227, *SE* = 0.150) than after performing the math task (*M* = 4.333, *SE* = 0.150; *p* < 0.001). Condition also significantly and independently predicted the relaxation score [*F* (2, 161.186) = 10.390, *p* < 0.001]. After controlling for running order and activity, relaxation scores were higher while participants viewed the tank containing live fish (*M* = 5.224, *SE* = 0.165) than when they viewed the tank containing plants and water (*M* = 4.562, *SE* = 0.165); *p* < 0.001), or when they viewed the empty tank (*M* = 4.553, *SE* = 0.165; *p* = 0.001). No other differences were statistically significant.

##### Mood Ratings

Activity significantly independently predicted mood [*F* (1, 169.103) = 28.322, *p* < 0.001], showing that mood rating was lower following the math task (*M* = 4.510, *SE* = 0.133) than following the aquarium viewing (*M* = 5.004, *SE* = 0.133; *p* < 0.001). Condition also independently predicted the mood ratings [*F* (2, 169.098) = 31.523; *p* < 0.001]. After controlling for running order and activity, ratings of mood were higher after participants viewed the tank containing the live fish (*M* = 5.249, *SE* = 0.140) than after they viewed the tank containing plants and water (*M* = 4.661, *SE* = 0.140; *p* < 0.001), which were also higher than after viewing the empty tank (*M* = 4.361, *SE* = 0.141; *p* < 0.001).

##### Comparison with Arrival/Baseline

HR and HRV were not measured upon arrival because the HR/HRV monitor was put in place following the completion of the initial paperwork (e.g., consent form, demographics and other questionnaires). Relaxation scores differed by condition [*F* (6, 210) = 12.353; *p* < 0.001]. Relaxation was higher after viewing the live fish (*M* = 5.857, *SE* = 0.202) than upon arrival (*M* = 5.714, *SE* = 0.202; *p* < 0.01]. Relaxation in the other conditions did not differ from relaxation at arrival. Mood scores differed by condition [*F* (6, 210) = 15.547, *p* < 0.001]. Mood was more positive (higher) after viewing the live fish (*M* = 5.543, *SE* = 0.157) than upon arrival (*M* = 4.857, *SE* = 0.157; *p* < 0.01). Ratings of mood were significantly less positive than arrival following the math task after viewing the empty tank (*M* = 4.114, *SE* = 0.157; *p* < 0.01). No other conditions were significantly different from arrival in terms of mood.

### 2.4. Discussion

The results of Experiment 1 showed that participants reported enhanced perceptions of mood and relaxation after viewing the live fish condition relative to the water and plants condition, the empty tank condition, and their mood and relaxation scores upon arrival at the test site. These results are consistent with our hypothesis, and are partially supported by the HR data, which showed a significant reduction in HR in the live fish condition relative to the plants and water condition, but curiously, not in the live fish relative to the empty tank condition. The HRV data did not vary significantly based on viewing condition. Taken together, these results indicate that perceptions of mood and relaxation are positively affected by viewing live fish, but the two physical measures of arousal (HR and HRV) did not demonstrate buffering as we predicted. If buffering had occurred, we would have expected to see reduced HR and HRV reactivity in the live fish condition relative to the other two conditions. It is possible that viewing live fish does not buffer these measures of arousal, or that HR and HRV were impacted by the stressor (the math task).

In this experiment, both HR and HRV were significantly impacted by the arousal associated with doing the math task, such that both measures indicated higher levels of arousal (HR was higher and HRV was lower) when participants were doing the math task than when they were viewing the aquaria. This may be because the math task was harder, and thus more arousing, but it could also be related to the fact that doing the math task required them to use their hand to write the answers on the sheet of paper, and viewing the tanks had not required any physical movement. That movement alone may have been enough to cause the changes we saw in the HR and HRV values.

Experiment 2 was designed as a partial replication, and extension, of Experiment 1. We wanted to replicate the basic finding that perceived relaxation was enhanced in the live fish viewing condition relative to the other two conditions, so we included the same three aquarium viewing conditions (live fish, water and plants, empty tank) and used the same relaxation scale. To examine the validity of the findings from Experiment 1, we wanted to include a measure that assessed a construct that is the opposite of relaxation and also employs a commonly-used scale. To achieve this goal, we added the State Trait Anxiety Inventory—Short form (STAI-S) which was used in research with undergraduate students [21,37].

While the math task did in fact increase HR and decrease HRV in Experiment 1, it is unclear if this was due to the challenging nature of the task or the physical activity required (hand writing and turning pages) by the task itself. We also were not sure that the math task increased arousal enough to be buffered by viewing the fish. In other words, there was very little social stress associated with the task in Experiment 1, because the problems were not scored in view of the participants. Additionally, the participants could view the entire set of math problems at once and answer only the easiest problems on the list in the time available. This very likely increased their scores and probably reduced their arousal. In Experiment 2, we changed the math task to a verbal face-to-face quiz, including immediate feedback as correct or incorrect. This change increased the difficulty level of the task by no longer allowing participants to select the easy problems at the exclusion of more difficult ones. It also added an increased level of social stress, because two experimenters were present and immediately aware of all math errors. Further, this new approach did not require the participants to move their hands or turn pages to answer the questions.

## 3. Experiment 2

As for Experiment 1, we predicted that relaxation would be enhanced following the viewing conditions relative to the math task and following the live fish condition relative to the plants and water, and the empty tank conditions. Similarly, for anxiety, we predicted that STAI-S scores would be lower after the viewing conditions than after the math task, and after the live fish condition compared to the other two conditions. We also predicted that HR and HRV would reflect lower levels of arousal in the live fish condition relative to the other two viewing conditions, and in the viewing conditions in general than during the math task.

As in Experiment 1, an indication that HR and HRV are sensitive to the arousal manipulation in this study (math task) will be demonstrated by a significant increase in HR, and a decrease in HRV, during the math task compared to the aquarium viewing conditions. If viewing live fish can buffer social stress from a verbal math task, then we expected HR to be lower, and HRV to be higher, in the live fish viewing condition than either of the other two conditions; plants and water, or the empty tank.

### 3.1. Method

#### 3.1.1. Participants

As was in Experiment 1, a priori sample size calculations indicated a sample size of 35 would provide a power of 0.80 with an alpha 0.05, correlations among repeated measures of 0.30 and a medium effect size. We over-enrolled the study in anticipation that some volunteers would not show up for their appointed data collection time slot, and as a result data were collected and used from 39 undergraduate students currently enrolled at the State University of New York at Fredonia. 

None of the participants in this experiment had also participated in Experiment 1. Six participants were male, 32 participants were female, and one participant identified as neither male nor female. Thirty-five participants self-identified as Caucasian, three identified as African American, one identified as Asian American, and two identified as Latino. Ages ranged from 18 years old to 22 years old (*M* = 19.43, *SD* = 1.32). The 0-to-4 scale GPA recorded by the participants ranged from a 2.1 to 4.0 (*M* = 3.41, *SD* = 1.04). Participants had zero to six (*M* = 0.95, *SD* = 1.36) older siblings and zero to four (*M* = 1.23, *SD* = 1.04) younger siblings. Two participants reported having an allergy to dogs, while 11 participants reported having an allergy to cats. Participants reported 0 to 7 close friends (*M* = 2.51, *SD* = 0.79). Fifteen participants reported that they currently resided with a pet, 11 of whom reported owning the pet. Twenty-four participants reported owning a pet that does not currently reside with them. The reasons they gave for the pet living elsewhere were: Nine participants indicated that their current housing did not allow pets, two responded that they did not have enough money to care for the pet, one responded that they did not have time to care for the pet, three responded they did not have enough room for the pet, nine responded that they knew the pet would not benefit living with them in their current living situation, one responded that their current roommate does not want a pet living with them, and eight participants listed reasons that were different than the ones provided.

#### 3.1.2. Materials

The same questionnaires were used in this experiment as were previously described in Experiment 1, except that the mood scale was not used. Instead, participants completed a six-item State-Trait Anxiety Inventory—Short form, or STAI-S [34]. Statements like “I feel calm”, “I feel upset”, and “I feel content” were presented with a corresponding 1–4 scale to the right of the statements, where participants would indicate to what extent they agreed with the given statement from Not at all, Somewhat, Moderately, to Very Much. The math task was also delivered differently. Instead of viewing and completing a full list of math problems, the same problems were shown one at a time in large print on 3” × 5” cards.

##### Aquaria and Apparatus

The same aquaria and equipment set-ups were used in this experiment as in Experiment 1. The fish included in the live fish tank condition were: One albino bristle nose Plecostomus (*Ancistrus temminckii*), 15 male fancy guppies (*Poecilia reticulata*), and seven emerald cory catfish (*Corydoras splendens)*. We opted to add the cory catfish to provide more movement at the bottom of the tank. Guppies tend to swim in the middle or surface layers of the water column, so the addition of the bottom dwellers made it very likely that wherever the students happened to look in the live fish aquarium, they were likely to see a fish.

#### 3.1.3. Procedure

The procedure for this experiment was the same as Experiment 1, except that the participants completed the STAI-S in place of the mood scale, and the math problems were presented one at a time by the experimenter, who then provided correct/incorrect feedback on the participant’s oral responses to the problems. A second experimenter was also present to keep track of time and to provide more social pressure by scoring the correct/incorrect responses.

### 3.2. Results

The best models for all outcomes included the main effects of activity and condition. For HRV the best model also included the main effect of running order as well as the interaction between running order and condition. For HR the best model also included the interaction between activity and condition. Best models for both relaxation and anxiety included the main effect of running order, but did not include any interactions.

#### 3.2.1. HRV

**Figure 1:**
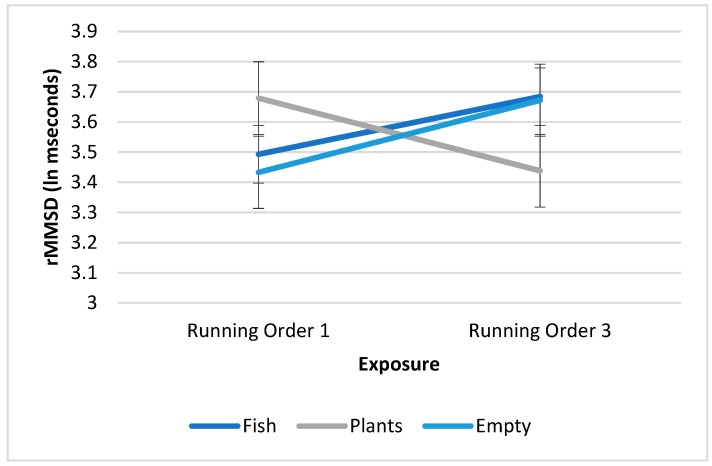
Average Heart Rate Variability (HRV) (looking at the three aquaria), when individuals looked at these aquaria as the first exposure to an aquarium and the third exposure to an aquarium (interaction *p* = 0.006).

The HRV data were transformed using a natural logarithmic transformation. HRV differed significantly and independently according to activity [*F* (1, 152.865) = 16.289, *p* < 0.001]. After controlling for condition, running order and the interaction of condition with running order HRV was lower while participants performed the math task (*M* = 3.486, *SE* = 0.080) than while they viewed the aquaria (*M* = 3.645, *SE* = 0.079; *p* < 0.001). The effect of condition was also significant [*F* (2, 172.076) = 4.828, *p* = 0.009], but none of the pairwise comparisons reached significance after controlling for multiple comparisons. Running order interacted significantly with condition (*p* = 0.006) and is displayed in Figure 1. The slopes of the trajectory of HRV from the first to the third exposure to the activities differed according to the condition. The slope of HRV is positive for the fish condition; HRV in the aquarium with fish condition is lowest when this is the first exposure to the two activities, and highest if this condition is the third exposure to the two activities. For the empty aquarium condition the slope is also positive. The opposite is true for viewing the tank with plants and water; the slope is negative. HRV in the empty aquarium condition is highest when this is the first exposure to the two activities, and lowest if this condition is the third exposure to the two activities (see Figure 1).

#### 3.2.2. HR

The HR data were winsorized at 115. HR differed significantly and independently according to activity [*F* (1, 190.00) = 14.601, *p* < 0.001]. HR was higher while participants performed the math task (*M* = 87.974, *SE* = 1.896) than while they viewed the aquaria (*M* = 82.289, *SE* = 1.896; *p* < 0.001). No other effects were statistically significant.

#### 3.2.3. Relaxation Score

Activity significantly independently predicted relaxation [*F* (1, 186.220) = 64.811, *p* < 0.001] after controlling for condition and running order. Relaxation was higher while participants viewed the aquaria (*M* = 5.482, *SE* = 0.171) than when they performed the math task (*M* = 4.547, *SE* = 0.173; *p* < 0.001). Condition also independently predicted relaxation [*F* (2, 184.327) = 7.183, *p* = 0.001]. After controlling for running order and activity, relaxation scores were higher in the live fish condition (*M* = 5.257, *SE* = 0.181) than in the empty tank condition (*M* = 4.728, *SE* = 0.181; *p* = 0.001). There was also a trend for relaxation to be higher in the plants and water condition (*M* = 5.059, *SE* = 0.181) than in the empty tank condition (*p* = 0.061). No other differences were statistically significant.

#### 3.2.4. Anxiety

Activity significantly independently predicted anxiety as measured by the STAI-S [*F* (1, 186.286) = 72.788, *p* < 0.001] after controlling for order. Anxiety rating was higher following the math task (*M* = 11.239, *SE* = 0.435) than after viewing the aquaria (*M* = 8.729, *SE* = 0.430; *p* < 0.001). Condition also significantly independently predicted anxiety rating [*F* (2, 184.371) = 9.256, *p* < 0.001]. Ratings of anxiety were lower after participants experienced the live fish condition (*M* = 9.221, *SE* = 0.456) than the empty tank condition (*M* = 10.756, *SE* = 0.456, *p* < 0.001). There was also a trend for anxiety to be lower after the plants and water condition (*M* = 9.973, *SE* = 0.457) than the empty tank condition (*p* = 0.088). No other differences were statistically significant.

#### 3.2.5. Comparison with Arrival/Baseline

HR and HRV were not measured upon arrival because the HR/HRV monitor was put in place following the completion of the initial paperwork (e.g., consent form, LAPS). Relaxation scores differed by condition [*F* (6, 234) = 15.805, *p* < 0.001]. Relaxation was higher after viewing the live fish (*M* = 5.744, *SE* = 0.197) than upon arrival (*M* = 4.923, *SE* = 0.197, *p* < 0.001). Relaxation was also higher after viewing the tank with plants and water (*M* = 5.615, *SE* = 0.197) than upon arrival (*p* = 0.006). Relaxation scores were lower following the math task in the empty tank condition (*M* = 4.33, *SE* = 0.197) than upon arrival (*p* = 0.039).

Anxiety also differed by condition [*F* (6, 234) = 16.523, *p* < 0.001]; anxiety was lower after viewing the live fish (*M* = 7.974, *SE* = 0.497) than it was upon arrival (*M* = 9.821, *SE* = 0.497, *p* < 0.001). Anxiety was also higher after viewing the empty tank and doing the math task (*M* = 11.308, *SE* = 0.497) than it was upon arrival (p = 0.001). There was also a trend towards higher anxiety scores after viewing the tank with plants and water (*M* = 5.615, *SE* = 0.197) than upon arrival (*p* = 0.055).

### 3.3. Discussion

The findings for the relaxation scale in the first experiment were replicated in Experiment 2 and are consistent with expectations based on the biophilia hypothesis. Extrapolating from the biophilia hypothesis, one would expect greater amounts of life or nature to produce more positive effects on the dependent measures than conditions involving less life or nature. That pattern was revealed in the results; viewing live fish plus plants and water was perceived as more relaxing than plants and water, which was more relaxing than the empty tank. Similar results were found with the measure of anxiety, which further supports the biophilia hypothesis. Interestingly, once again these significant differences among the viewing conditions were not apparent in the HR or HRV data. However, an interaction with running order indicates that the order in which a participant is exposed to the different viewing conditions can impact their HRV during the viewing conditions. When they saw the plants and water condition first, their HRV was high (indicating relaxation), but when it was the third exposure, their HRV was significantly lower. The opposite trend was apparent in the live fish and empty tank conditions, indicating that the first exposure is less relaxing than the third. These findings suggest that future research should attempt to tease out the running order and time course of these effects on HRV, as both could have introduced enough variability into the data to conceal the impact of the viewing conditions on these measures.

Relaxation scores were higher and anxiety was lower when comparing these measures from arrival to after viewing the live fish, suggesting that viewing live fish improves both of these measures. Similar comparisons for HR and HRV were not possible, because the monitor was added after the participants completed the initial paperwork, including reading and signing the consent form.

The results indicate that the math task successfully served the purpose of increasing participants’ arousal/stress levels, and that all measures were sensitive to this change. Relaxation scores were lower, anxiety scores were higher, HR was higher and HRV was lower, all indicating higher levels of arousal or stress around performing the math task, when compared to viewing the aquaria. 

These findings indicate that the math task was sufficiently arousing/stressful to be detected by our dependent measures. However, the predicted interactions with viewing condition were not present in the data. If viewing live fish buffered this type of arousal/stress, then we would have seen the measures reflect that buffering, by showing higher relaxation and HRV and lower anxiety and HR during the math task when it followed the live fish viewing, relative to the other two viewing conditions. This leaves us to conjecture that the lack of significance may indicate that viewing live fish is not an effective buffer from this type of stressor, or that the measures were not sensitive enough to detect the presence of such effects.

## 4. Limitations

It is possible that self-report measures like mood, relaxation and anxiety are vulnerable to demand characteristics in that the participants may think they should have enhanced mood and relaxation and reduced anxiety when viewing the live fish condition, and so they score these measures accordingly. Self-report measures, such as mood, have been specifically explored for their vulnerability to demand characteristics, and the results show that although some of the variance may be due to demand characteristics, variation in these measures are not just artifactual [38].

It is somewhat concerning that HR and HRV did not consistently match the pattern of perceived enhanced relaxation, mood and reduced anxiety in the live fish condition, but it is possible that physiological levels of arousal may not reflect the perceived changes. It is also possible that this study may be underpowered to detect the changes, or that those changes may take more exposure time to be revealed in the HR or HRV data. Another potential explanation for the absence of the anticipated changes in HR and HRV is that caffeine intake among individuals in the study participants’ age group tends to be above average, and caffeine intake can impact these measurements [39]. Further research is required to flesh out whether live fish can buffer stress as measured by HR and HRV, or if other measures of stress (e.g., cortisol) are required to detect any stress buffering impact of viewing live fish.

## 5. General Discussion

The two experiments reported here consistently showed that viewing live fish in an aquarium, as compared to the other two viewing conditions (plants and water, or empty tank) and participants’ condition upon arrival, enhances self-reported relaxation and mood, and decreases anxiety. These findings partially replicate and extend previous research on this topic, and it is the first study to include the range of measures used herein in a single study. For example, one study involving university students who were about to give a speech, examined anxiety as measured by the STAI [21]. The results showed that five minutes of viewing live fish in an aquarium reduced anxiety relative to the control condition. Another study showed that videos of animals (including fish) produced lower HR than watching a popular soap opera [22]. Yet another study showed that participants spent more time viewing fish tanks when they had more, rather than fewer, fish in them [23], but none of these studies have included a manipulation of the amount of “life” in a tank while measuring multiple relevant dependent measures.

As described earlier, the results of this study are largely, but not completely, consistent with predictions based upon the biophilia hypothesis, in that enhancements in dependent measures were seen in the viewing conditions with the most “life” or nature (live fish, plants and water) when compared to those with less “life” or nature (plants and water or empty tank). The biophilia hypothesis is conceptually too broad to provide concise predictions, but if one were to extrapolate from the basic hypothesis, it would seem likely that stress should have been buffered in the live fish condition relative to the empty tank condition, and that interaction was not significant in either experiment.

According to the biopsychosocial model, we may see improved mood, relaxation and reduced anxiety in the live fish condition because the fish may provide a form of social support via companionship, an implied opportunity to nurture or provide care, a reduction in loneliness, or a distraction from perceived stressors. 

However, it seems likely that this model would also predict similar effects to be present in the HR and HRV data across viewing conditions, such that HR should be lower and HRV higher in the live fish condition relative to the empty tank condition. This is partially supported by the HR findings in Experiment 1, but not in Experiment 2, and not by any of the HRV findings.

Arguably the most parsimonious explanation for the findings of these two experiments is distraction, where the live fish function to draw the participants’ attention away from perceived stressors. This would explain why we see enhancements in the self-report measures, but do not consistently see similar effects in HR and HRV. Participants are able to take their minds off stressors temporarily, but may not be able to achieve a high enough level of distraction to experience corresponding physiological effects as measured by HR and HRV. To fully explore this idea, further research is needed involving a larger manipulation of distraction carried out over a longer time, to determine whether more time is needed to realize the physiological effects of distraction. It is also possible that HRV measurements may have been affected by outside factors such as caffeine intake, and thus may not be the best measure of stress reduction in this population. Cortisol, for instance, may provide a better assessment.

## 6. Conclusions

The results of these experiments provide partial support for both the concept of biophilia and the biopsychosocial model, particularly with respect to self-reported improvements in mood and relaxation. That these improvements were not consistently discernible physiologically by measurements of HR and HRV, may reflect demand characteristics inherent in such self-report measures, or they may be the result of other factors which could be controlled for in future experiments. The fact that this study demonstrated that individuals perceived benefits including a decrease in stress, elevation of mood, and increase in relaxation while viewing a tank of live fish, that they did not experience with a tank with plants but no fish, or a tank with neither plants nor fish, provides a promising foundation for additional research.
